# Immune Microenvironment of Thyroid Cancer

**DOI:** 10.7150/jca.44506

**Published:** 2020-06-08

**Authors:** Hongyu Yin, Yemei Tang, Yujia Guo, Shuxin Wen

**Affiliations:** 1 Shanxi Key Laboratory of Otorhinolaryngology Head and Neck Cancer, Shanxi Medical University, Taiyuan 030001, Shanxi, P.R. China.; 2 Department of Otolaryngology Head & Neck Surgery, The First Hospital of Shanxi Medical University, Taiyuan 030001, Shanxi, P.R. China.; 3 Shanxi Province Clinical Medical Research Center for Precision Medicine of Head and Neck Cancer, The First Hospital of Shanxi Medical University, Taiyuan 030001, Shanxi, P.R. China.; 4 General Hospital, Shenzhen University, Shenzhen 518061, Guangdong, P.R. China.

**Keywords:** Thyroid Cancer (TC), Immune Microenvironment, Immune Evasion, Immunotherapy

## Abstract

Thyroid cancer (TC) is a highly heterogeneous endocrine malignancy with an increased incidence in women than in men. Previous studies regarding the pathogenesis of TC focused on the pathological changes of the tumor cells while ignoring the importance of the mesenchymal cells in tumor microenvironment. However, more recently, the stable environment provided by the interaction of thyroid cancer cells with the peri-tumoral stroma has been widely studied. Studies have shown that components of an individual's immune system are closely related to the occurrence, invasion, and metastasis of TC, which may affect response to treatment and prognosis of the patients. This article presents a comprehensive review of the immune cells, secreted soluble mediators and immune checkpoints in the immune microenvironment, mechanisms that promoting TC cells immune evasion and existing immunotherapy strategies. Besides it provides new strategies for TC prognosis prediction and immunotherapy.

## Introduction

Thyroid cancer (TC) is the most prevalent endocrine malignancy, and its incidence has been increasing in the recent three decades. The prevalence is reported to be higher in females than in males 1. The incidence of all types of thyroid cancers has increased and treatment depends on the type and how far it has spread. Four main types of thyroid cancer are distinguished based on histological features: papillary thyroid carcinoma (PTC), follicular thyroid carcinoma (FTC), medullary thyroid cancer (MTC) and anaplastic thyroid carcinoma (ATC). The four differ in tumor aggressiveness, progression, treatment, and prognosis. PTC and FTC are the most common thyroid cancers; they have low overall mortality but are more likely to relapse. Even though MTC and ATC are rare, they are characterized by high invasiveness, early metastasis and poor prognosis 2, 3. The current conventional treatment methods for TCs are surgery, radiotherapy, and endocrine suppression treatment. However, with these methods, it is difficult to obtain satisfactory curative effects 4, 5. One of the possible explanations is that the tumor interacts with the surrounding immune microenvironment during its occurrence and development, and this mediates immune tolerance and tumor escape.

In 1970, Burnet and Thomas 6 proposed the theory of “immune surveillance”, which suggested that the immune system can recognize and eliminate infected cells that may be transformed into cancerous cells and protect against invasion of adjacent tissues. In 2002, Dunn et al. 7 proposed the theory of “tumor immunoediting” supporting the broader perspective of immune surveillance. Based on this theory, the interaction between the tumor and immune system is divided into three stages: elimination, equilibrium, and escape. The elimination phase is an equivalent of immune surveillance. During the equilibrium phase, tumor cells begin to mutate and can escape the immune response. In the escape phase, the tumor cells escape from the immune control and this plays a critical role in tumor progression. Some studies have suggested that the tumor escape the immune system through “camouflage and destruction” 8. Camouflage refers to the reduction of the expression of major histocompatibility complex (MHC) class I molecules, and escape killing by cytotoxic T lymphocytes (CTLs) and other cells. To defend themselves against the body's immune response, several immune cells such as myeloid suppressor cells and regulatory T cells are recruited where they promote tumor growth in a process referred to as destruction.

More than 100 years ago, Stephan Paget proposed the “seed and soil” hypothesis and his observations were that tumor cells act like seeds and metastasis depends on the surrounding microenvironment. The hypothesis is subsequently accepted and confirmed 9, 10. The tumor microenvironment involves not only cytogenetics but also an understanding of tumor behavior in the surrounding microenvironment 11. Recently, some scholars have reported that the immune status of the TC microenvironment is closely related to patients' prognosis. Researchers have attempted to change the immune microenvironment of TC through immunotherapy to make up for the deficiency of surgery and radiotherapy 12. This article describes the relationship between the major functional immune cells, related soluble factors and immune checkpoints in the TC immune microenvironment. Besides, the article also describes the occurrence, development, metastasis, and prognosis of TC, which provides the theoretical basis for the development of new targets for immunotherapy and prognosis prediction.

## Immune cells in TC

### T cells

T lymphocytes are derived from pre-lymphoid progenitor cells in the bone marrow that mature in the thymus and mediate cellular immune responses. They are categorized into three types based on immune effector functions: regulatory T cells, cytotoxic T cells, and helper T cells.

#### Regulatory T Cells

Regulatory T cells (Tregs) refer to a wide range of T cells that are capable of recognizing T cell receptor (TCR)-antigen peptides presented by MHC molecules in target cells and exert certain immunosuppressive function. There are many types of Tregs and among these CD4^+^CD25^+^Tregs are the most physiologically important. Tregs are highly enriched in tumor microenvironment where they limit the anti-tumor immune response 13. A large number of Tregs infiltrating in tumors are associated with poor prognosis of patients 14, 15. Therefore, focusing on how Tregs migrate, recruit and accumulate in the tumor microenvironment and how they modulate these aspects may help control tumor progression and reduce tumor resistance to treatment.

Currently, the mechanism of Tregs migration, recruitment, and accumulation in tumors is being explored. Some studies have suggested that regulatory T cells can efficiently migrate into tumors in response to chemokines expressed on the stroma and tumor cells. Tregs are known to accumulate in tumor tissues and this is mainly promoted by CC chemokine receptor 4 (CCR4)-CC motif ligand 17/22 (CCL17/22), CCR10-CCL28, and CXC chemokine receptor 4 (CXCR4)-CXC motif ligand 12 (CXCL12). The secretion of immunosuppressive factors such as transforming growth factor (TGF)-β, vascular endothelial growth factor (VEGF), and indoleamine2,3-dioxygenase (IDO) have modulatory effects on Tregs 16. FoxP3+T cells are preferentially attracted to the tumor microenvironment with the help of chemokines and this transforms them to not only highly proliferative but also highly transformative.

CD4^+^CD25^+^T cells are reported to be significantly more abundant in patients with PTC and multinodular goiter (MNG) than in MNG patients 17, making PTC more invasive and with a poor prognosis. The underlying mechanism could be that plasmacytoid DCs (pDCs) in the PTC microenvironment induces the transformation of initial CD4^+^T cells into FoxP3^+^ICOS^+^Tregs through the inducible costimulatory ligand (ICOSL)-ICOS pathway hence resulting in Tregs enrichment and tumor escape. Cunha et al. 18 used immunohistochemistry to detect the expression of FoxP3 in 253 cases of PTC and 13 cases of FTC and compared them with benign thyroid lesions and normal tissues. FoxP3 expression was found to be associated with the aggressiveness of differentiated thyroid carcinoma. Besides, it was also reported that the smaller the tumor diameter the higher expression of Foxp3. Similarly, Liu et al. 19 found that Tregs content in peripheral blood and tumor tissues of PTC was higher than that in MNG patients, which was closely related to extra-glandular invasion and lymph node metastasis (LNM). The poor prognosis indicated that FoxP3 may be a predictor of TC and a molecular target for future immunotherapy.

#### Cytotoxic T Cells

Cytotoxic T lymphocytes (CTLs) are mainly differentiated from CD8^+^T cells and possess the ability to specifically kill target cells. They are characteristic markers of good prognosis in some tumors, such as colorectal cancer and ovarian cancer 20, 21. Previous studies reported that patients with high-density CD8^+^CTLs show better survival rates and lower biological behaviors which are manifested as low T stage and absence of metastasis. CD8^+^T cells are an important part of tumor adaptive immune response and they are restricted by their own MHC class I molecules. Upon activation, CD8^+^T cells bind to tumor target cells and secrete interferon (IFN)-γ. Consequently, IFN-γ performs a series of immunosuppressive behaviors such as inhibiting cell proliferation, promoting apoptosis and inducing macrophages to devour tumor cells and protect the host 22.

French et al. 23 found that in PTC patients, the low concentration of CD8^+^T cells was low or a reduced ratio of CD8/Foxp3^+^T cells is associated with a larger tumor diameter. Further, a reduced number of CD8^+^T cells weakens the lethality to tumor cells and facilitates the rapid growth of tumor cells and strengthens their invasiveness. Xiang et al. 24 screened the CLDN10 gene by using bioinformatics methods. They reported that the gene was related to lymph node metastasis (LNM) of PTC but was a predictor of a good prognosis. Further analysis found that CLDN10 increased CD8^+^T cell infiltration. Therefore, the role of CD8^+^T cell infiltration on prognosis should be considered in cancer research.

#### Helper T Cells

Helper T (Th) cells mainly differentiate from activated naïve CD4^+^T cells. Differentiate Th cells possess different effects, secrete different cytokines, and exert different immune effects. Th1 enhances and expands the cellular immune response process by secreting interleukin (IL)-2, IFN-γ and other cytokines and induces other immune cells to exert anti-tumor effects. Th2 suppresses the anti-tumor effect of cellular immunity by secreting factors such as IL-4, inhibits the activation of natural killer (NK) cells, and reduces the expression of tumor cell antigens 25. Th1/Th2 is a good indicator of the dynamic changes during the body's anti-tumor immunity.

### Natural Killer (NK) Cells

Natural killer cells were discovered in the late 1960s. They can exert their effects even without prior stimulation and kill target cells. They are a component of the innate immune system hence play a role in the first line of the host immunological defense 26. A balance between the activation and inhibition of receptors on the surface of NK cells determines the outcomes of their interactions with target cells 27. NK cells destroy pathogenic cells mainly by secreting perforin, granzyme and express Fas ligand (FasL) and destroy their targets through antibody-dependent cell-mediated cytotoxicity (ADCC) 28. Studies have shown that the infiltration of NK cells in tumors tends to predict a good prognosis 29. However, in the tumor microenvironment, the function of NK cells is limited. Studies have reported that tumor cells secrete immunosuppressive factors such as TGF-β, IDO, and arginase-1, which reduce the expression of NK cell surface activated receptors and result in a decrease in the number and quality of NK cells. Tumor cells reduce the expression of MHC class I molecules, interrupt the tumor antigen-presenting pathway, and evade immune surveillance. Tumor cells evade NK cell attacks through several mechanisms such as upregulation of inhibition ligands, stimulation of inhibitory regulatory T lymphocytes and shedding of activating ligands 27.

In PTC, NK cell infiltration is reported to be more enhanced than in MNG, and the infiltration in the early stages of the disease is higher compared to that in advanced stages 30. This suggests that NK cells play a certain regulatory role in PTC. Studies have found that in advanced TCs such as ATC, the frequency of CD56^hi^/CD16^hi/lo^ NK cells increases with an increase in programmed cell death protein 1 (PD-1), T cell immunoglobulin and mucin domain-3 (TIM-3). PD-1 and TIM-3 blockade is effective at reinvigorating cytotoxicity of CD56^hi^/CD16^hi/lo^ NK cells, suggesting that ATC mediates NK cells dysfunction through the PD-1/TIM-3 pathway in the immune microenvironment 31. Moreover, activated receptors such as natural killer group 2, member D (NGK2D) receptors are reported to be decreased in the immune microenvironment of TC, leading to the inhibition of NK cell activity and loss of normal function and tumor escape 32. Therefore, it is important to regulate NK cells receptors and consider the use of PD-1/TIM-3 as targets for tumor immunotherapy.

### Mast Cells (MCs)

Mast cells are bone marrow-derived immune cells. They circulate as immature precursors in peripheral blood circulation system and develop into functional mast cells after entering the peripheral tissues 33. MCs are important effector cells of the innate immune system and regulators of adaptive immune responses 34.

Studies have shown that MCs act like a double-edged sword in tumors 35. They secrete vascular endothelial growth factor (VEGF) to promote tumor angiogenesis, secrete matrix metalloproteinases (MMPs) to facilitate tumor invasion, and secrete cytokines such as tumor necrosis factor (TNF)-α and IL-8 to stimulate immune tolerance and promote tumor progression. In cutaneous melanoma 36, 37, researchers have found that MCs are increased with the pathological progression of melanomas and this is accompanied by an increase in fibroblast growth factor 2 and VEGF, indicating that mast cells promote tumor progression. MCs also release cytokines such as IL-10, IL-2, IFN-γ which play a direct anti-tumor role. Macrophages and basophils are recruited around the tumor to indirectly exert cytotoxic effects on tumor cells and inhibit tumor progression. These phenomena have been manifested in many solid tumors, such as colorectal cancer and the five-year survival rate is reported to be significantly higher in patients with higher MCs infiltration, suggesting that MCs might inhibit tumor growth 38.

The role of MCs has been widely studied in thyroid cancer and its density has been positively correlated with cancer aggressiveness 39. Some researchers injected MCs into the tail vein of mice and found that the growth rate of mice transplanted tumors increased. Mast cell-derived histamine, chemokine CXCL1/growth regulating oncogene 1 (GROα) and CXCL10/interference inducible protein 10 (IP10) are reported to accelerate the proliferation and invasion of thyroid cancer cells *in vitro* 40. However, these specific effects can be reversed by MC inhibitors. Visciano et al. 41 found that TC cells activate MCs to produce chemokines, which induce epithelial-mesenchymal transition (EMT) and stem cell features in TC cells. The molecular pathway involved maybe that of IL-8 phosphorylates Akt pathway which promotes the expression of Slug protein. Blocking of this pathway or Slug protein inhibition can reverse EMT and stemness, thereby inhibiting the invasion of TC cells. Therefore, the mast cells-dependent CXCL8/IL-8-Akt-Slug pathway can be considered as a target in the development of treatment modalities for thyroid cancer.

### Tumor-associated Macrophages (TAMs)

Tumor-associated macrophages are the most abundant population of tumor-infiltrating immune cells in TME and have strong plasticity. They are categorized into two cell subgroups: the M1 classical type, mainly produces cytokines such as TNF-α and IL-1 which inhibit and kill tumor cells; the M2 alternate type produces factors such as IL-10 and IL-13 which promotes the occurrence and development of tumors 42.

TAMs are “an accomplice in the progression of solid tumors”. They do so by remodeling the extracellular matrix, facilitating angiogenesis, inhibiting immune activity, and promoting tumor cell migration and invasion 43. TAMs play an extraordinary role in tumor development and progression. In the TME, cancer cells secrete signaling factors to induce monocyte macrophages to differentiate into M2 type through extracellular vehicles (EVs). This causes an imbalance of the M1/M2 ratio hence promoting cancer development 44. In lung cancer, the M1/M2 ratio is reported to be significantly related to patients' overall survival. M1 type significantly inhibits tumor growth and angiogenesis, while M2 type enhances its invasion ability 45. In oral squamous cell carcinoma, CD163-labeled macrophages (M2 type) are reported to induce epithelial-mesenchymal transition (EMT) of cancer cells which increases their aggressiveness. Besides, the patient's overall survival rate is significantly reduced, indicating that M2 type macrophages infiltration significantly correlates with poor tumor prognosis 46.

In thyroid cancer, the products secreted by activated macrophages are affected by cancer cells and immune cells in the tumor microenvironment and these play a role in cancer progression and invasion. Qing et al. 47 used immunohistochemistry to detect the expression of CD68-labeled macrophages in PTC and benign lesions. They found that the macrophages infiltration rate in PTC was significantly higher than that in benign tumors, and also positively correlated with lymph node metastasis and poor prognosis. Fang et al. 48 found that the TAMs density was directly proportional to the invasive characteristics of PTC. Also, the cytokine array analysis revealed that the mechanism may be associated with CXCL8 derived from TAMs binding to CXCR1/2 secreted by PTC cells to promote cancer cell metastasis. Selective removal of TAMs inhibits the growth of PTC, and colony-stimulating factor 1 (CSF-1)/CSF-1R signals can be used as pharmacological targets to weaken the growth of BRAF^V600E^-induced PTC 49. Targeted combination therapy using BRAF inhibitors likely to produce a good response in advanced TCs. Studies have revealed that CCL15 is the most abundant chemokines in FTC compared with follicular adenoma (FA). This important feature is of great significance in the preoperative differential diagnosis of the two diseases 50. However, due to limitations of sample size, TAMs have only been shown to play an early role in FTC and the correlation between TAMs and clinicopathological characteristics in patients has not been found and requires further research. In conclusion, high TAMs density in TC is closely associated with tumor invasion and reduced survival. Therefore, there is a need to fully understand the functional differences of different subtypes of TAMs in the thyroid gland to provide a deeper understanding of the balance between cancer promotion and suppression. Besides, this is important for considerations of TAMs as specific targets in the treatment of thyroid cancer.

### Dendritic Cells (DCs)

Dendritic cells are the main types of professional antigen-presenting cells in the body 51. They link the innate and adaptive immune responses. However, in recent years, the antitumor effect of DCs in TME has remained controversial and they have been associated more with tumor immune escape. This is because DCs mainly exist in an immature status and secrete some suppressive cytokines such as TGF-β, IL-6, etc. that silence the immune response 39.In pancreatic ductal adenocarcinoma models, DCs are reported to be inhibited by Tregs, are unable to express costimulatory molecular ligands, and are unable to activate CD8^+^ T cells required to produce antitumor effects 52.

TC cells can recruit dendritic cells. The interaction between the Met/Hepatocyte Growth Factor (HGF) in the PTC microenvironment stimulates the release of macrophage inflammatory protein (MIP)-1α which recruits DCs expressing the homologous receptor CCR6 to facilitate the clearance of damaged thyroid cells 53. In PTC, the infiltration density of CD1a-labeled DCs in tumors is increased and results in improved disease-free survival (DFS) to a certain extent 54. However, some studies have found that there is no significant correlation between S100-labeled DCs density and DFS 55. This is due to the differences in the enrichment sites of CD1a+ and S100+ phenotypes in tumors 56. Other studies have found that both DCs and Tregs increase in PTC because DCs induce the transformation of CD4^+^T cells to FoxP3^+^Tregs. The interaction between DCs and Tregs is an important mechanism for tumor escape in PTC 17. Therefore, disrupting the interaction between the two can be considered as a new treatment strategy for TC (**Table 1**).

## Soluble mediators in TC

Soluble mediators include a large group of polypeptide molecules produced by several types of cells, including cytokines, chemokines, angiogenic factors, etc. which play an important role in the initiation and development of thyroid cancer 57, 58.

### The role of cytokines/chemokines in TC

Interleukin (IL)-1 has been found to promote tumor angiogenesis and metastasis 59. It is highly expressed in several cancers and predictor of poor prognosis 60. The difference in IL-1β serum concentration between atrophic thyroiditis and PTC is often used to discriminate between the two diseases 61. IL-10 is produced by TAMs and malignant cells and has multiple effects on both innate and adaptive immunity which is mainly to as immunosuppressive effect 62. Studies have shown that IL-10 is expressed in both benign and malignant thyroid diseases, but the expression is higher in TC. This suggests that an increase in IL-10 promotes malignant transformation. Moreover, the expression of IL-10 is related to extra-thyroidal invasion and large tumor size 63. Stassi et al. 64 found that IL-4 and IL-10 increase the resistance of TC cells to chemotherapy through the up-regulation of anti-apoptotic proteins such as Bcl-2/xL. In PTC patients, serum IL-6 levels are reported to be higher compared to healthy controls, and preoperative IL-6 levels are higher than those after surgery 65. In an ATC mouse xenograft model, it was reported that IL-12 with anti-tumor activity can be used to treat a subcutaneous tumor, and the mechanism involved is dependent on NK/NK-like cells to release perforin which kills tumor cells 66.

Angiogenesis is a major feature of tumors 67. Although the main source of VEGF is MCs, almost every TC-infiltrating immune cell can produce VEGF 68-71.

Chemokines attract leukocytes to the site of inflammation 72. Chemokines are classified into four main subfamilies: CXC, CC, C, and CX3C. Specific chemokines and their specific receptors may be involved in the development of TC 73, 74. TCs expressing tumor proteins RET/PTC and BRAF often express high levels of chemokines, such as CXCL8, CXCL12, CCR2, and CCR20 75. In PTC, CXCL12 was reported to be related to angiogenesis and metastasis. Furthermore, using CXCL12 as a diagnostic marker, the sensitivity and specificity were reported to be more than 90% 76. CCL2/MCP-1 (monocyte chemotactic protein 1) promotes lymph node metastasis in PTC patients by recruiting TAMs which express CCR2. Besides, patients with higher CCL2 expression are more likely to experience recurrence 77. CCL20 is also referred to as macrophage inflammatory protein-3 (MIP-3) and it is the ligand for CCR6 78. PTC patients express higher levels of CCL20 and CCR6 relative to healthy controls 79. Treatment with TNF-α significantly increases the migration of TC cells expressing CCR6 and all these make TCs more aggressive 80.

CXCL8 is an immunological landmark that is widely expressed in several human malignancies 81. CXCL8 promotes cancer metastasis mainly by recruiting neutrophils. Inoue et al. 82 found that in transitional epithelial cancer, CXCL8 (IL-8) regulated tumorigenicity and metastasis by upregulating matrix metalloprotein 9 (MMP-9) expression. The involvement of CXCL8 in epithelial-mesenchymal transition confirms its ability to promote metastasis 83. Both normal human thyrocytes and tumor thyroid cells can secrete CXCL8. CXCL8 mainly plays a vital role in autoimmune thyroid diseases and in maintaining thyroid homeostasis 84. Studies have shown that the ability of TPC-1 (RET/PTC) and BCPAP (BRAF^V600E^ mutation) cell lines to secrete CXCL8 is significantly different since oncogene types also differ in cancer cells 85. Previous studies suggest that high levels of CXCL8 in tumors are positively correlated with TC aggressiveness an indication that tumor progression can be inhibited by lowering CXCL8 levels *in vivo* 86. Such pharmacologic strategies have been used in the treatment of tumors either alone or combine with other treatment approaches 87.

### Pharmacologic strategies of lowering CXCL8

Biguanide drugs not only treat diabetes but are also known to have certain anti-tumor effects. Metformin significantly inhibits TNF-α induced CXCL8 secretion in DTC and thus inhibits the growth of cancer cells achieved by activation of the AMPK pathway 88, 89. Phenformin is reported to inhibit CXCL8 secretion 90. Adenosine monophosphate analogue 5-aminoimidazole4-carboxamide ribonucleotide (AICIR) is another AMPK activator that also inhibits CXCL8 secretion and prevents CXCL8-induced TC cells migration. Slightly different from biguanides, it can not only affect CXCL8 secretion in normal human thyrocytes (NHT) cells but also CXCL8 secretion in TPC-1 and BCPAP cancer cell lines 91. Reparixin is an allosteric inhibitor of CXCR1/2 (CXCL8 receptors) which significantly decreases TC cell proliferation, epithelial-mesenchymal transition, survival, and more importantly stemness. This suggests that reparixin treatment not only inhibits tumor metastasis but also reduces chemoresistance and improves on the response rate 92. The oncolytic adenovirus dl922-947 has been previously shown to destroy tumor neovascularization in the immune microenvironment and promote TAM to M1 type transformation. This depends on the transcription factors Nuclear Factor-kappa B (NF-κB) and P65 which reduces the production of CXCL8 and MCP-1 in ATC cell lines 93. Coperchini et al. 94 found that the BRAF^V600E^ mutation is associated with more aggressive behaviors in TC and enhances the secretion of CXCL8. The BRAF-inhibitor PLX4720 made the concentration of CXCL8 more or less decreased in three cell lines of BRAF^V600E^ (BCPAP, 8305C and 8505C), and also inhibited the CXCL8-induced cell migration. Currently, BRAF inhibitors have only shown clinical benefits in melanoma, and have not been used in TC therapy. Besides, drug resistance has bottlenecked the treatment to some extent and this is reported to be associated with the components changes in the tumor microenvironment 95.

## Immune checkpoints in TC

Immune checkpoints are regulators of T cell immune response. Similar to the braking system of T cells, they can limit the immune system's attack on healthy cells and prevent the occurrence of autoimmune diseases 96. Immune checkpoints contain co-stimulatory and inhibitory molecules, both of which regulate the immune system. Negative immune checkpoints including cytotoxic T lymphocyte antigen 4 (CTLA-4), programmed cell death protein 1 (PD-1), programmed cell death ligand 1 (PD-L1), and indoleamine 2,3-dioxygenase (IDO), etc. inhibit the activity of T cells to kill tumor cells and promote tumor cells the escape 97. The expression of these factors is reported to be common in TC cells.

PD-1/PD-L1 pathway has become an important component of tumor immunosuppression. The two pathways interact with each other to inhibit cytotoxic T lymphocytes and enhance tumor proliferation and invasion 98. In ATC, PD-1 expression was reported on the surface of inflammatory cells and PD-L1 expression in tumor cells. Both were associated with worse progression-free survival (PFS) hence can be used as prognostic markers 99.

Ahn et al. 100 studied PD-L1 using immunohistochemistry in 407 cases of TC and found that the expression of PD-L1 varied in different cases. The positive expression rates in PTC, FTC, and ATC were 6.1%, 7.6%, and 22.2% respectively. Further, PD-L1 was highly expressed in advanced thyroid cancer and since ATC was extremely invasive, palliative surgery was considered. To improve the patient's quality of life, it is important to find corresponding immunotherapy targets. Although PD-L1 is not common in ATC, it can be associated with its treatment. In another study, limited PD-L1 expression was reported in MTC 101.

In PTC, the expression levels of CTLA-4 and PD-L1 were inversely correlated to the thyroid differentiation score (TDS) and were reported to be higher in tumors having BRAF^V600E^ mutations 12.

Moretti et al. 102 found that the IDO mRNA level was higher in various thyroid cancers than in normal tissues and that the expression levels were positively correlated with aggressiveness. Interestingly, in PTC, IDO expression showed a significant correlation with FoxP3, which suppressed the immune microenvironment by inducing the regulation of the FoxP3 phenotype to promote tumor escape. Similarly, in papillary thyroid microcarcinoma (PTMC), the positive expression rate of IDO was 31% and this was positively correlated to FoxP3 expression and also closely associated with aggressive characteristics such as extrathyroidal extension and multifocality 103.

## Immune escape of TC cells

### Down-regulation of MHC class I molecules

MHC class Ⅰ molecules are mainly responsible for the presentation of tumor-associated antigens (TAAs) on the surface of tumor cells. The expression of these cellular antigens and immune co-stimulatory signals not only promotes the activation of the immune system but also kills abnormal cells. The absence of MHC class I molecules on the tumor cell surface is recognized as an important mechanism for tumor immune escape 104. Angell et al. 105 found that compared with normal thyroid tissue, the expression of human histocompatibility antigen (HLA)-ABC and β2 microglobulin in PTC was significantly reduced, and the proportion of tumor-infiltrating lymphocytes (TILs) was also decreased. This phenomenon can be reversed through the intervention of interferon and selumetinib on the constructed cell model to increase the number of HLA-ABC, β2 microglobulin, and TILs. Therefore, the absence of MHC class I molecules is an important escape strategy for TC cells.

### Up-regulation of B7 Homolog 1 (B7-H1)

B7-H1 is a member of the B7 family which is also known as programmed death-ligand 1 (PD-L1). The binding of B7-H1 and its receptor PD-1 inhibits T cell proliferation and certain cytokines secretion *in vitro*, which also exist as negative costimulatory molecules in the process of T cell activation 106. B7-H1 induces CTLs apoptosis which is the main mechanism for mediating tumor escape. In PTC, B7H1 protein and mRNA levels increase and are closely associated with tumor aggressiveness. The higher the B7H1 expression level the stronger the tumor aggressiveness 107. Shi et al. 108 reported that in MTC, PD-L1 was associated with invasive pathological features such as larger tumor diameter, lymph node metastasis, and later tumor stage, while PD-L1 expression was higher in patients experiencing a recurrence. These help TC evade immune surveillance and play a vital role in normal cell transformation into tumor cells.

### Up-regulation of Hypoxia Inducible Factor (HIF)-1α

The accelerated proliferation rate and the poorly formed blood vessels in tumor cells increase oxygen consumption resulting in hypoxic. Hypoxia stimulates the proliferation of tumor cells and angiogenesis 109. Besides, it activates the tumor STAT3 signal transduction pathway, promotes the synthesis of downstream HIF-1α and production of vascular endothelial growth factor (VEGF), is involved in the activation of regulatory T cells and also possesses an immune-suppressive effect which promotes tumor escape 110.

Hypoxia induces increased expression of HIF-1α and PD-L1 in FTC which is closely associated with later TNM stage and distant metastasis 111, 112. Also, HIF-1α and PD-L1 inhibition delay tumor progression and formation. Koperek et al. 113 found that HIF-1α was beneficial to the remodeling of tumor stroma in PTC, and was significantly related to lymph node metastasis and extravascular invasion. This feature is also present in MTC 114. In conclusion, high expression of HIF-1α promotes the proliferation and transformation of thyroid cancer cells, evades immune surveillance, and can be used as a potential therapeutic target 115.

## Immunotherapy strategies for TC

**Figure 1:**
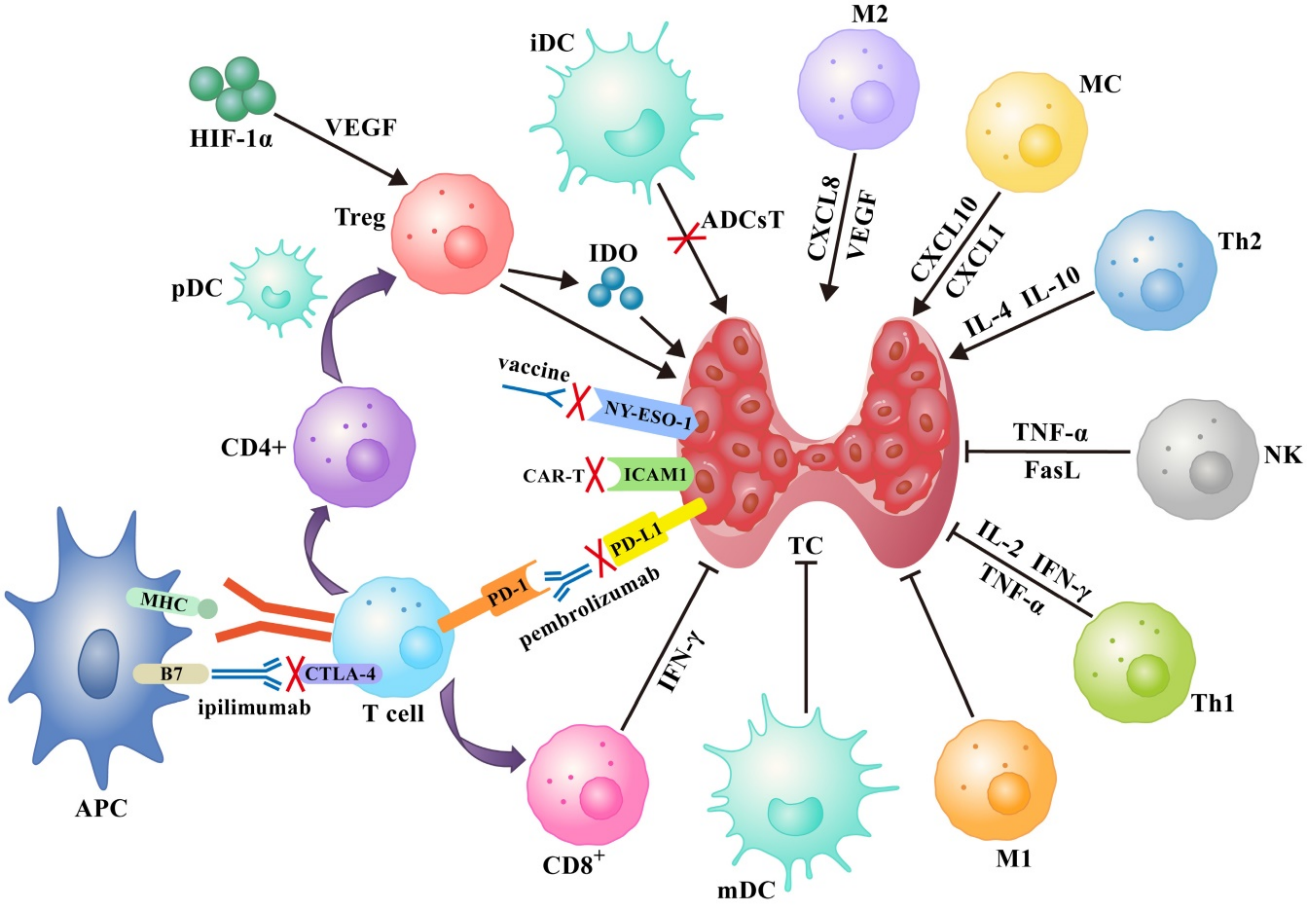
A schematic diagram of the immune network for thyroid cancer contains immune cells, immune checkpoints and several soluble factors that work together, as well as idealized treatments based on this such as tumor vaccines, immune checkpoint inhibitors, and adoptive cell therapies (see text for details). **Abbreviations:** ADCsT: adoptive dendritic cells therapy; APC: antigen-presenting cell; iDC: immature dendritic cell; mDC: mature dendritic cell.

Several immunotherapy strategies have been developed based on the immune response to specific antigen peptides present on tumor surfaces or in the cytoplasm. Treatments such as tumor vaccines and adoptive cell therapy increase the ability of body cells to kill tumors. On the other hand, immune checkpoint inhibitors (ICIs) improve the ability of immune cells to suppress tumor development (**Figure 1**).

### Tumor vaccines

Several tumor-associated antigens (TAAs) and neoantigens with strong immunogenicity have been identified on the surface of TC cells. These antigens are potential targets for immunotherapy. Cancer/Testis antigens (CTAs) are present on the surface of MHC class I molecules. They are recognized by cytotoxic T lymphocytes expressed in various malignant tumors 116. For instance, the New York esophageal squamous carcinoma 1(NY-ESO-1) which belongs to the CTAs families has been widely used as a target for tumor vaccine immunotherapy. Clinical trials have tested the performance of NY-ESO-1 targeted immunotherapy in melanoma 117 and esophageal cancer 118. Despite these trials, the clinical benefits of NY-ESO-1 vaccine and note fully understood and deserve further exploration.

Previously, NY-ESO-1 was detected in 15 of 23 MTC cases, and antibodies against NY-ESO-1 were detected in the serum of such patients. This showed that MTC is accompanied by humoral responses and immunogenicity. Moreover, it was also found that NY-ESO-1 contributed to the recurrence of MTC as revealed by Computed Tomography (CT) tests. This suggests that NY-ESO-1-based vaccines may be effective for MTC treatment 119, 120.

### Adoptive Cell Therapy (ACT)

Adoptive cell therapy refers to treatments that are based on immunocompetent cells isolated from tumors. These cells are expanded and functionally characterized *in vitro*. When injected *in vivo*, they can kill tumor cells directly or stimulate immune responses to indirectly kill tumor cells.

Previously, tumor lysate-pulsed DCs were inoculated into patients with advanced PTC, FTC 121 and MTC 122. Results showed that this therapy was well-tolerated and the body mounted effective immune responses to attack tumor cells. Some of the patients showed symptoms remission, indicating that DCs immunotherapy is effective for advanced thyroid cancer.

In addition to non-specific immune cell therapies such as DCs, other specific cell therapies such as chimeric antigen receptor (CAR)-T cell therapy have been developed. CAR-T cell therapy targeting intercellular adhesion molecule (ICAM)-1 was preclinically validated in TCs, especially in PTC and ATC 123, 124. ICAM-1 increased the sensitivity of tumor cells to CAR-T cells and thus killed tumor cells. CAR-T cell therapy yielded marked elimination of tumors and prolonged survival rate in mice and humans. However, further studies are required to optimize the ability of T cells to uptake and to enter tumors, while reducing side effects and overcoming the immunosuppressive state brought by CAR-T cell therapy 125.

### Immune Checkpoint Inhibitors (ICIs)

Immune checkpoints inhibitors have been shown to enhance the body's anti-tumor immunity. They are therefore widely used to treat various tumors, including TC. Several immune checkpoint inhibitors have been put on the market such as CTLA-4 monoclonal antibodies (as ipilimumab), anti-PD-1 monoclonal antibodies (as pembrolizumab) and anti-PD-L1 monoclonal antibodies.

Mehnert et al. 126 tested the efficacy and safety of pembrolizumab in patients with advanced PTC and FTC with positive PD-L1 expression. They found that these drugs were well tolerated in patients. Among the 22 subjects, 2 showed significant symptoms of remission, and no significant adverse events or death were recorded, indicating that this therapy is safe. A combination of anti-PD-1 and tyrosinase inhibitors decreased the tumor metabolic activity and prolonged the survival rate of some patients with ATC expressing PD-L1 127. Lenalidomide eliminated the inhibitory effects of the immune checkpoint on immune responses 128. To date, several drug combinations have been developed to treat advanced thyroid cancer, with many of them showing no adverse reactions 129. Mutations in BRAF gene have been identified as the cause of poor prognosis of ATC. BRAF^V600E^ was found to inhibit the immune microenvironment of tumors. Therefore, in murine ATC, the combination of BRAF inhibitor PLX4720 and anti-PD-1/PD-L1 antibody was found to not only inhibit tumor growth, prolong the survival time of mice, but also reshape the immune system and improve the quality of life 130.

## Outstanding Questions and Conclusions

Thyroid cancer is the most common malignant tumor affecting head and neck regions and the endocrine system. It accounts for about 2% of all systemic malignancies 131. In recent years, the incidence of thyroid cancer is the fastest among all malignancies in China 132. TC has several histological types and heterogeneous prognosis 2, 3, 133, 134. TC utilizes several complicated mechanisms to escape the body's immune system. Numerous immune cells infiltrate the TC microenvironment, which is characterized by several multilevel communications among various cancer cells, cytokines, chemokines, angiogenic factors, etc. These factors influence patients' outcomes 135.

Some studies have shown that TAMs and MCs promote the progression of TC. TAMs are the most infiltrating inflammatory cells around tumors, accounting for about 50% of all immune cells. In the TC microenvironment, TAMs mostly exist as M2 type and are closely correlated to more malignant biological behaviors, such as larger tumor diameter 48, lymph node metastasis 136, and distant lung metastasis 137, worse TC differentiation, and higher the density of TAMs. VEGF produced by TAMs provides a suitable basis for TC infiltration and metastasis through increasing vascular permeability. During the progression of TC, MCs are recruited around the tumor, and the density of MCs is positively correlated with tumor proliferation and invasive capacity 138. However, other subtypes of TAMs have been identified apart from M1, M2 139. MCs and macrophages found in the myeloid system are of dual hematopoietic origin 140, implying that MCs may contain several subtypes that are yet to be discovered. We propose further single-cell RNA sequencing investigations about macrophages and MCs subsets in the development of TC. This will lead to the identification of biomarkers for treatment of TC.

NK cells are important immune cells in the body with the power to kill tumor cells. Wennerberg et al. 32 found that NK cells in ATC tumors are significantly compromised compared with those in peripheral blood due to decreased NGK2D expression. They also found that patients with high UL-binding protein (ULBP)2/5/6 expression were sensitive to NK cell-mediated pyrolysis, suggesting that ULBP2/5/6 may be a biomarker for NK cell immunotherapy. TC cells influence the prognosis of patients by altering the phenotype of NK cells 141, but the mechanisms underlying this role require further exploration. Regulatory T cells are T cells that control autoimmune reactivity in the body and induce immune tolerance by releasing anti-inflammatory factors IL-10, TGF-β, etc. 142. DCs are the strongest antigen-presenting cells in the body. In TC, DCs do not mature effectively in the local microenvironment 143. This leads to the production of inhibitors, such as TGF-β and angiogenesis factor VEGF, which inhibit T cell immune function, promote angiogenesis and facilitate tumor growth 87.

Since TC cells and immune cells secrete some pro-tumorigenic chemokines, cytokines and angiogenic factors, decreasing the expression of these soluble mediators may be of therapeutic value in TC. CXCL8 is the most studied chemokine. Suppression of CXCL8 decreases the aggressiveness of TC as revealed in several pharmacological studies 89-94.

Accumulating evidence shows that TC and thyroid autoimmunity often co-exist and they co-stimulate inflammation and increase the expression of transcription factors such as NF-κB 144. NF-κB induces the secretion of chemokines, cytokines, and other molecules in the tumor microenvironment. Zeng et al. 145 reported that CCL20/CCR6 induces MMP-3 production through the NF-κB pathway and promotes the invasion and metastasis of TC cells. Similarly, Bauerle et al. 146 observed that NF-κB promotes TC cells invasion by driving MMP-9 transcription to some extent. NF-κB inhibitors promote TNF-α-mediated apoptosis of TC cells through continuous activation of the c-Jun N-terminal kinase (JNK) pathway. These results indicate that immunotherapy targeting NF-κB may be useful for the treatment of thyroid cancer.

Tumor immunotherapy is based on the principle that the immune system can accurately distinguish between normal cells and cancer cells. This function is dependent on the ability of MHC class I molecules to present antigens on the surface of tumor cells to immune cells in time. However, tumor cells can subtly reduce the expression of MHC class I molecules, making them unable to bind tumor-associated antigens, thus avoiding the recognition and killing of immune cells 147. High expression of B7-H1 molecules inhibits the receptor PD-1 on the surface of T cells, further suppressing the activity of T cells, an effect that promotes immune escape of tumors 107. Hypoxia is an important feature of solid tumors. HIF-1α is a key transcription regulating molecule that mediates the adaptive response of tumor cells to the hypoxic microenvironment. It activates regulatory T cells, upregulates VEGF, suppresses immune responses, and promotes angiogenesis and tumor invasion.

Although ICIs consistently induce immune responses in tumors, the response rate is not high. Thus, the focus of clinical research should be directed towards improving the efficacy of ICIs 148. Immune checkpoint inhibitors are associated with adverse events. Endocrine disorders are approximately 29% of adverse events 149. Therefore, thyroid function should be monitored regularly during the treatment process. If patients develop adverse reactions, the drug should be discontinued in time. Similarly, other tumor treatment strategies, such as vaccines and adoptive immune cell therapies, show weak efficacy against tumors. Combination immunotherapy strategies are proposed as they may provide effective tumor control 150. Each biomarker related to TC growth and progression may be considered as a potential target for immunotherapy (**Table 2**).

This study describes the immune microenvironment of TC, focusing on immune cells related to cancer progression and related molecules that promote cancer immune escape. The data reviewed here provide a deeper understanding of the pathogenesis and metastasis mechanisms of TC. (Figure 1) The potential immune cells and soluble mediators that predict treatment responses as well as strategies for improving immune defense against tumors are discussed. We hope the ideas presented in this review will stimulate further research into the discovery of strategies to suppress cancer immune escape and other immunotherapy solutions.

## Figures and Tables

**Table 1:** Role of immune cells in TC microenvironment

Immune cells	TC type	Methods used	Reported data	Reference
T cells	PTC	IHC/IF/FC	Tregs and pDCs together contribute to the tumor escape in patients with PTC plus MNG.	17
	PTC/FTC	IHC	Foxp3 is highly expressed in differentiated thyroid cancer and is associated with aggressiveness.	18
	PTC	IHC/FC	TC cells activate Tregs to evade immune attack, suppressing the host's immune response.	19
	PTC	IHC/IF	TAL and low CD8/Treg ratios are associated with more aggressiveness in PTC.	23
	PTC	bioinformatics	CLDN10 increases the infiltration of CD8+T cells and predicts favorable prognosis in PTC.	24
NK cells	PTC	FC	FC analysis showed significantly increased NK cells in PTC tissue compared with MNG tissue and is negatively correlated with advanced disease.	30
	ATC	FC	ATC mediates the dysfunction of NK cells through the PD-1/TIM-3 pathway.	31
	ATC	IHC/IF	ATC cell lines are sensitive to NK cell-mediated lysis via ULBP2/5/6 and chemoattract CXCR3-positive NK cells.	32
MCs	PTC	Xenografts	MCs can be recruited into the tumor site in vivo and enhance cancer cell proliferation.	40
	PTC/ATC	WB	Mast cells, activated by TC cells, produce IL-8 that induces EMT via Akt signaling.	41
TAMs	PTC	IHC	The high density of TAMs is associated with LNM, possibly through the secretion of CXCL8.	47,48
	PTC	IHC/FC	PTC requires CSF-1/CSF-1R signaling to recruit TAMs as it progresses.	49
	FTC	IHC/CKAbA	FTC may induce TAMs infiltration by producing CCL15, which may be applied clinically to differentiate FTC from FA pre-operation.	50
DCs	PTC	Migration Assay	HGF stimulates TC cells to release chemokines active in recruiting DCs	53
	PTC	IHC	Increased density of DCs infiltration may improve DFS, or may not be significantly correlated.	54,55
	PTC	IHC/IF/FC	DCs induce Tregs transformation and promote the escape of PTC.	17

**Abbreviations:** CKAbA: cytokine antibody array; FC: flow cytometry; IHC: immunohistochemistry; IF: immunofluorescence; TAL: tumor-associated lymphocytes; WB: western blot.

**Table 2:** Summary of the prognostic values of biomarkers in TC

Biomarker	Prognostic value					
	Invasiveness	Differentiation grade	Tumor stage	Metastasis	Clinical survival	Response to immunotherapy
IL-10	+ 63	N/A	N/A	N/A	N/A	N/A
CCR6	N/A	N/A	N/A	+ 80	N/A	N/A
CXCL8	N/A	N/A	N/A	+ 86	N/A	N/A
CTLA-4	N/A	-[12]	N/A	N/A	N/A	N/A
PD-1	N/A	N/A	N/A	N/A	-[98]	N/A
PD-L1	+ 107	-[12]	N/A	+ 108	-[98]	PD-1 inhibition: + 127
IDO	+ 103	N/A	N/A	N/A	N/A	N/A
MHC I	-[105]	N/A	N/A	N/A	N/A	N/A
HIF-1α	N/A	N/A	+ 111	+ 112	N/A	N/A
NY-ESO-1	N/A	N/A	N/A	N/A	N/A	Vaccination: + 119,120
DCs	N/A	N/A	N/A	N/A	N/A	Adoptive DCs therapy: + 121,122
ICAM-1	N/A	N/A	N/A	N/A	+ 84	CAR-T: + 123

+, positive correlation; -, negative correlation; N/A, not available.
